# Identification of a Proanthocyanidin from *Litchi Chinensis* Sonn. Root with Anti-Tyrosinase and Antioxidant Activity

**DOI:** 10.3390/biom10091347

**Published:** 2020-09-21

**Authors:** Matthew Saive, Manon Genva, Thibaut Istasse, Michel Frederich, Chloé Maes, Marie-Laure Fauconnier

**Affiliations:** 1 Laboratory of Chemistry of Natural Molecules, Gembloux Agro-Bio Tech, University of Liège, 4000 Liège, Belgium; Chloe.Maes@uliege.be (C.M.); marie-laure.fauconnier@uliege.be (M.-L.F.); 2 Biomass and Green Technologies, Gembloux Agro-Bio Tech, University of Liège, 4000 Liège, Belgium; thibaut.istasse@uliege.be; 3 Laboratory of Pharmacognosy, Center for Interdisciplinary Research on Medicines (CIRM), University of Liège, 4000 Liège, Belgium; m.frederich@uliege.be

**Keywords:** proanthocyanidins, *Litchi chinensis*, anti-tyrosinase activity, DPPH, molecular identification

## Abstract

This work follows an ethnobotanical study that took place in the island of Mayotte (France), which pointed out the potential properties of *Litchi chinensis* Sonn. roots when used to enhance skin health and appearance. Through in vitro testing of a crude methanolic extract, high anti-tyrosinase (skin whitening effect) and antioxidant activities (skin soothing effect) could be measured. HPLC successive bio-guided fractionation steps allowed the purification of one of the compounds responsible for the biological activities. The isolated compound was characterized by UV, IR, MS and 2D-NMR, revealing, for the first time in *Litchi chinensis* Sonn. roots, an A-type proanthocyanidin and thus revealing a consensus among the traditional use shown by the ethnobotanical study, in vitro biological activities and chemical characterization.

## 1. Introduction

### 1.1. Context

An ethnobotanical study which took place in the island of Mayotte [1] led to the conclusion that many plants are used traditionally among inhabitants of the island to promote health and for use in dermatological applications. Using indicators such as the Informant Agreement Ratio developed by Trotter & Logan, 1986 [2], species of interest were selected for further studies. In vitro biological activities measurements realized on several crude extracts of the selected plants revealed that *Litchi chinensis* Sonn. roots, traditionally used for skin whitening and soothing, have high anti-tyrosinase and antioxidant activities.

### 1.2. Litchi

The species *Litchi chinensis* Sonn. which belongs to the *Sapindaceae* family [3,4] mainly originates from southeast Asia but is now a cultivated economic crop in the countries around the world with appropriate climate for its culture [5,6]. The *Litchi* Sonn. genus only contains one identified species composed of three subspecies: *L. chinensis* subsp. *chinensis* Forest & Kim Starr, *L. chinensis* subsp. *phippinensis* Radlk, and *L. chinensis* subsp. *javensis* Leenh. [7,8]. The first one is mainly found in China [4], the second subspecies is native from the Philippines, New Guinea, Malay Peninsula, and Indonesia and the last one is endemic from Java. Due to their commercial interest, *L. chinensis* subsp. *chinensis* Forest & Kim Starr and *L. chinensis* subsp. *phippinensis* Radlk are found all around the Indian ocean [3]. In Mayotte, only the subspecies *chinensis* is encountered [9].

**Figure 1:**
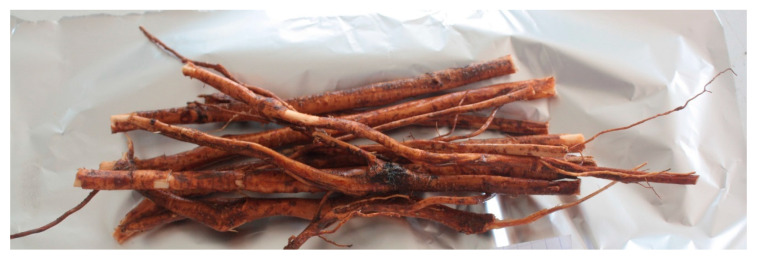
Picture of *Litchi chinensis* roots. (Credit Matthew Saive).

*L. chinensis* is an evergreen, medium sized round-topped tree. Its leaves are leathery, pinnate or lanceolate, acuminate and glabrous. Its inflorescence is a branched panicle. The flowers are small going from white to pale yellow, with a tetramerous calyx without a corolla. They are functionally male and female. The litchi fruits are heart shaped to round covered by a rough rind of pericarp going from light pink to red. The root system is strongly influenced by the soil as well as the propagation technique used. Even if some specimens present a tap root, most of the *Litchi* trees found nowadays have fibrous roots (Figure 1) found at depth between 0 and 60 cm [4,10,11].

In addition to its use as food, many work have pointed out the biological, including medicinal, properties of *L. chinensis* [12] which are gathered in Table 1.

### 1.3. Anti-Tyrosinase Activity

Skin pigmentation is due to melanogenesis, a biochemical phenomenon that takes place in the basal layer of the epidermis. The melanins are produced there and provide the skin with natural protection against harmful effects of UV rays. Melanogenesis can be disturbed due to external aggressions like UV, hormonal disturbances or aging and may result in the appearance of hyperpigmentation spots. The cosmetics sector is actively looking for compounds capable of inhibiting tyrosinase activity with a view to develop ethnic or anti-spot/anti-aging cosmetics [14].

Ethnobotanical study and preliminary anti-tyrosinase and antioxidant in vitro activity measurements incited us to further study *Litchi chinensis* root extracts by purifying and characterizing a compound involved within the observed biological activities.

## 2. Materials and Methods

DMAC (4-dimethylamincinnamaldehyde), L-DOPA (3,4-dihydroxy-L-phenylalanine), kojic acid, formic acid (FA), K_3_PO_4_, TROLOX (6-hydroxy-2,5,7,8-tetramethylchroman-2-carboxylic acid), DPPH (2,2-diphenyl-1-picrylhydrazyl), tyrosinase (E.C. 1.14.18.1) from mushroom, ammonium formate were purchased from Sigma Aldrich (Darmstadt, Germany). NaH_2_PO_4_ was purchased from Merck (Darmstadt, Germany) and Na_2_HPO_4_ from UCB (Bruxelles, Belgium). All HPLC grade and technical solvents as well as absolute ethanol used in this work were purchased from VWR (Leuven, Belgium).

### 2.1. Plant Material

During the ethnobotanical study, *Litchi chinensis* roots were collected around the village of Coconi, (Mayotte Island, 12°50′03.3″ S 45°08′24.2″ E) and a specimen was stored into the CBNM’s herbaria for proofing reasons (referenced as MAO00051). A total of three independent collects occurred between 2014 and 2017 on trees of similar size in the same area. The *L. chinensis*’s roots were dried for 48 h at 40 ± 1 °C using a drying oven, they were then powdered, vacuum packed and stored at −22 ± 1 °C until being used. Extractions and purifications were therefore carried out on three completely independent biological samples.

### 2.2. Sample Preparation

Twenty grams of *L. chinensis* roots were precisely weighted and extracted with 400 mL of solvent in a Soxhlet apparatus. Extractions were made using either HPLC grade methanol or HPLC grade dichloromethane, and this led to the collection of a polar and a less-polar extract. The extracts were then evaporated at 40 ± 1 °C using a rotating evaporator (Sigma Aldrich, Darmstadt, Germany). The dried extracts were kept at −22 ± 1 °C until use. The polar samples were solubilized in a small amount of pure ethyl alcohol and the non-polar extracts were solubilized in HPLC grade dimethyl sulfoxide. The solvents were added gradually under ultrasonic bath until complete solubilization.

### 2.3. DPPH Antioxidant Activity

In order to determine the antioxidant potency of the plant samples, a protocol based on the DPPH method and adapted from M. S. Blois 1965 [15] was used. A 2.10^−4^ M DPPH solution was prepared using methanol and was then added to different dilution of the plant extracts. The solution was left to react for 30 min and then observed at 517 nm by UV/VIS spectrophotometer (Ultrospec 7000 from Biochrom, Cambridge, UK). The polar plant extract was diluted using HPLC grade methanol and the non-polar extract was diluted using HPLC grade ethyl acetate. The 10^−2^ and 10^−3^ dilutions were applied to the plant extracts. A 1:1 (v/v) ratio was applied with the reagent and the plant samples. The positive control both kind of samples was TROLOX (6-hydroxy-2,5,7,8-tetramethylchroman-2-carboxylic acid) 2.10^−3^ M in methanol and blank was 100% methanol.

### 2.4. Tyrosinase Inhibition

Tyrosinase (EC 1.14.18.1) is a key enzyme in the melanogenesis pathway. In this work, the polyphenol oxidase activity was exploited based on the adaptation of the work of Rangkadilok et al., 2007 [16]. Through the consumption of L-DOPA, it produces dopachromes that are detected/measured at 475 nm. In order to evaluate the impact of the plant extract on the synthesis of these dopachromes, inhibitions were compared with the inhibitory action of 1 mM kojic acid in phosphate buffer (0.05 M–pH 6.5). Dilution factors of 10^−1^, 10^−2^ and 10^−3^ were applied to the samples using the same phosphate buffer. The substrate solution was made of a 1 mM solution of L-DOPA in distilled H_2_O and the enzymatic suspension was made at a 625 U/mL concentration in phosphate buffer. As oxygen is also part of the enzymatic reaction as a co-substrate, the phosphate buffer used in the reactive mix was saturated with pure oxygen for 30 min at room temperature prior to the analysis.

The enzymatic reaction was performed by mixing 100 µL of sample with 50 µL of tyrosinase and 250 µL of oxygenated phosphate buffer. This mixture was left to incubate for 15 min, and then added in a quartz absorption cell along with 400 µL of the substrate solution. The synthesis of dopachromes was observed in real time, and the absorbance was measured every 15 s for 5 min at 475 nm.

### 2.5. Bio-Guided Fractionation

Throughout the purification process, the anti-tyrosinase inhibition test and the antioxidant test were conducted for all fractions. Based on preliminary observation, the chromatograms were systematically recorded at 280 nm as it gave the best contrast; however, in order to control potential left out compounds, other wavelengths were also registered (254 nm, 310 nm and 360 nm) with the cut-off for eluants being at 190 nm.

#### 2.5.1. Preparative HPLC

A first preparative HPLC step was conducted as follows: the column was a 150 × 21.2 mm–5 µm RP C8 column (Strategy, Interchrom, Interchim, Montluçon, France). Two mL solution (100 mg/mL) of the samples was injected at a 20 mL/min flow. The mobile phase was composed of solvent A: acetonitrile (CAN)–0.1% formic acid and solvent B: H_2_O–0.1% formic acid, and the optimized gradient was from 100% B to 20% (A)–80% (B) in 50 min, and then a final hold for 5 min. The mobile phase reached 60% (A)–40% (B) at 65:00 min and was then reset to 100% (B) in 10 min. From this first cycle, 17 fractions were collected and tested for the two biological activities. The most potent fraction went through a second preparative HPLC purification process.

The second preparative HPLC was conducted on this fraction with the same apparatus; the detector was set at 280 nm; 2 mL of sample at a 100 mg/mL were injected at a 16 mL/min flow rate. The instrument was fitted with a 250 × 22 mm–10 µm RP C4 column (Protein from Vydac, Hicrom, Lutterworth, UK). The same solvents were used for this fractionation. The gradient started with a steady step with 0% (A)–100% (B) between the 00:00 min mark and the 05:00 min mark. At the 10:00 min mark, it had reached 5% (A)–95% (B). At the 55:00 min mark, it had gone down to 30% (A)–70% (B); from that point it went back up to 0% (A)–100% (B) at the 57:00 min mark. From this second cycle, 5 fractions were collected and tested for the two biological activities. The most potent fraction was once again fractionated using an analytical HPLC setup mounted with a fraction collector.

#### 2.5.2. Purification through Analytical HPLC

A Hewlett-Packard Agilent 1200 HPLC (Santa Clara, CA, USA) with a Multi Wave detector set at 280 nm was used, and the following parameters where applied. Ten microliter of 10 mg/mL sample was injected at a 0.5 mL/min flow rate. The solvent used were the same as the one used for the preparative HPLC. The instrument was equipped with a 250 × 4.6 mm–5 µm RP C8 column (Strategy from Uptisphere, Interchim, Montluçon, France). The gradient starts at 0% (A)–100% (B) for 10 min; then from the 10:00 min mark to the 55:00 min mark, it goes down to 20% (A)–80% (B); this is followed by a drop reaching 60% (A)–40% (B) at the 57:00 min mark. It finishes at the 59:00 min mark with a 0% (A)–100% (B) mobile phase. From this fractionation 5 fractions were collected and tested for the two biological activities. Once the purification process was complete, the compound was kept at 7 ± 1 °C in a dark container for a maximum 48 h before undergoing the analytical steps.

### 2.6. Molecular Characterization

The molecular characterization of the target compound was realized by combining IR, UV, 2D-NMR and MS techniques.

#### 2.6.1. IR

The dry sample was analyzed using a Shimazu IR Affinity-1S FTIR (Nakagyo, Japan) spectrophotometer in transmission mode. The purified powder was placed directly on the diamond in order to establish its IR spectrum. The FT-IR spectrum of the sample was scanned between 4000 and 400 cm^−1^.

#### 2.6.2. UV

The UV spectra was obtained with a Ultrospec 7000 (Biochrom, Holliston, MA, USA) spectrophotometer. The sample was diluted at a 0.3 mg/mL in the MeOH and the absorbance was recorded in the UV/VIS range between 200 to 600 nm.

#### 2.6.3. MS

The purified fraction of interest was solubilized in HPLC grade MeOH at a 1 mg/mL concentration. It was analyzed using an Agilent 1100 HPLC (Santa Clara, CA, USA) mounted with an Inertsil ODS-3 3 µm 3 × 100 mm column and using the following gradient: acetonitrile: mQ water (85:15, solvent B) and mQ water (solvent A) + 0.2% formic acid and 12 mM ammonium formate. One minute of 53% B was followed by a linear increase of B to 100% at 17 min and an isocratic elution at 18 min. The gradient was then reversed at 6 min followed by a stabilization after 3 min at a flow rate of 0.5 mL/min. The injected volume was 10 µL.

Then using an ESI-ion trap mass spectrometer (Esquire HCT ion trap mass spectrometer; Bruker, Rheinstetten, Germany), the fraction of interest was characterized. The ESI was operated in the positive mode. Mass spectrometer parameters were set as follows: capillary voltage -4500 V; endplate offset −500 V; nebulizer pressure 50 psi; dry gas flow rate 10 L/min; dry gas temperature 300 °C; skimmer 20.6 V; capillary exit 300 V; oct 1 DC 10 V; oct 2 DC 2.79 V; Lens 1–5.2 V; lens 2–72 V; oct RF 300 Vpp; trap drive 92.8. The scan range was optimized at 300–1250 m/z.

#### 2.6.4. NMR

The purified sample was diluted in D_2_O with TSP as internal standard and 2D (^1^H–^13^C) NMR spectra were recorded at 300 K on a Bruker Avance NEO Ultrashield 700 Plus equipment operating at 700 MHz for ^1^H and 175 MHz for ^13^C and using a TCI cryo-probe (Rheinstetten, Germany).

For heteronuclear single quantum correlation (HSQC) experiments, the datasets were acquired with 1024 and 512 data points for the f2 (^1^H) and f1 (^13^C) dimensions, with spectral widths of 11161 and 38736 Hz, respectively. Eight scans were performed and the relaxation delay was fixed at 1 s.

Regarding heteronuclear multiple bond correlation (HMBC) experiments, the datasets were acquired with 4096 and 512 data points for the f2 (^1^H) and f1 (^13^C) dimensions, with spectral widths of 9091 and 38736 Hz, respectively. Thirty two scans were performed and the relaxation delay was fixed at 1.5 s.

#### 2.6.5. Colorimetric Test

In order to confirm the properties of the component, a colorimetric test based according to Prior et al., 2010 [17] was performed. The DMAC (4-dimethylaminocinnamaldehyde) test has proven to be effective when trying to quantify proanthocyanidins in plant extracts [18]. A 0.1 mg/mL sample solution was prepared in pure ethanol. The DMAC solution was done following Prior’s protocol: 0.01 g of DMAC was placed in a 10 mL volumetric flask and a solution of 36% hydrochloric acid, distilled water and pure ethanol (1:1:6) was added to the mark. The reaction was performed at room temperature: 100 µL of sample solution was placed in a spectrophotometric cell added with 500 µL of DMAC solution and read immediately at 640 nm.

## 3. Results and Discussion

### 3.1. Species Selection

Twenty-one species were selected after the ethnobotanical study in Mayotte (France) and their crude extracts obtained with methanol and dichloromethane were used for biological in vitro testing (data not shown). Among the 21 species tested, *Litchi chinensis* was the most promising, exhibiting high anti-tyrosinase and antioxidant activities (95.1 ± 0.06%, 84.8 ± 1.87%, respectively).

### 3.2. Bioguided Fractionation

**Figure 2:**
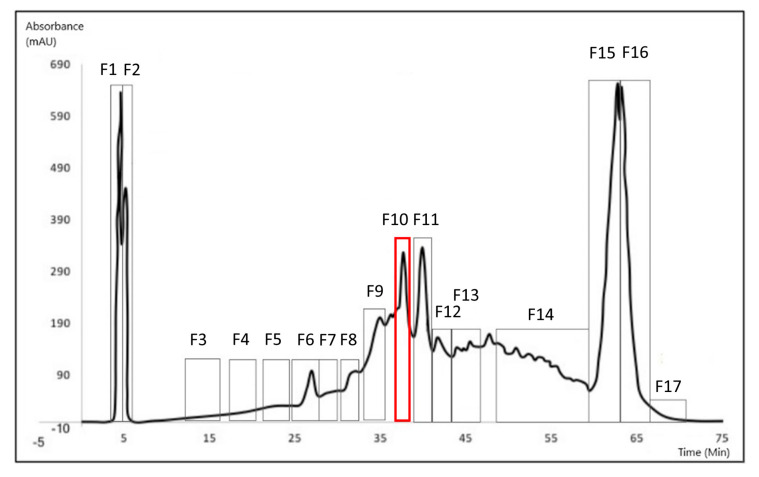
Chromatogram from the first fractionation process (preparative HPLC). Seventeen fractions were isolated. The peak of interest is F10 (RT = 37′49″) (shown in red).

**Figure 3:**
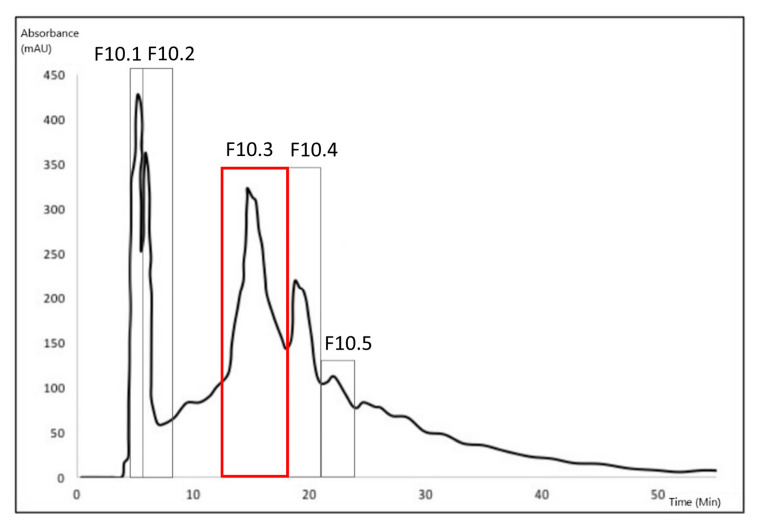
Chromatogram from the second fractionation process (preparative HPLC). Five fractions were isolated. The peak of interest is F10.3 (RT = 14′45″) (shown in red).

**Figure 4:**
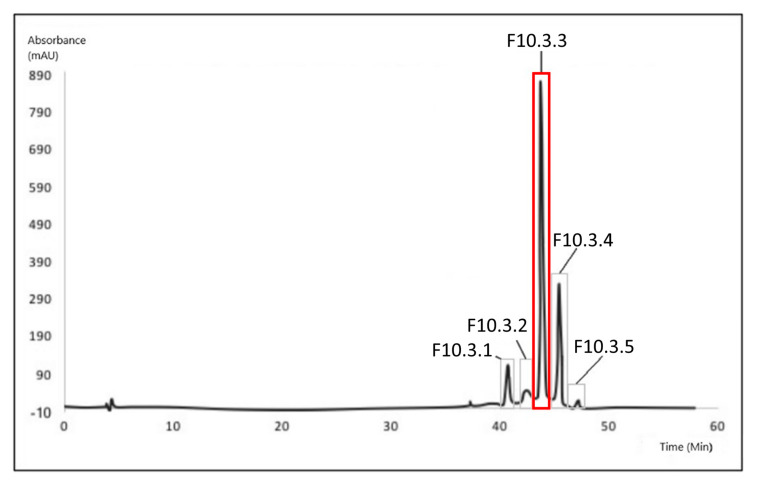
Chromatogram from the third fractionation process (analytical HPLC with fraction collector). Five fractions were isolated. The peak of interest is F10.3.3 (RT = 43′49″) (shown in red).

The bio-guided successive fractionation steps are illustrated in the different chromatograms shown below (Figure 2, Figure 3 and Figure 4). In each chromatogram, the peak that was selected after each fractionation and biological activities evaluation (Table 2, Table 3 and Table 4) has been pointed out. In the first, fraction F10 was selected (Figure 2) because of its biological activities (Table 2); in the second one, F10.3 was selected because of its biological activities (Table 3) (Figure 3). The identification work took place on peak F10.3.3 (Figure 4), and its biological activities can be found in Table 4, obtained after the third fractionation/purification step.

The Table 2, Table 3 and Table 4 present the relative anti-tyrosinase and antioxidant activities of each fraction collected respectively after one, two or three HPLC purification step(s). In Table 2, the fraction 10 is highlighted for its high anti-tyrosinase and antioxidant activities and was further fractionated into sub-fractions (Table 3) where fraction 10.3 was selected for the third fractionation step (Table 4). The molecular characterization took place on fraction 10.3.3.

**Table 2:** Relative activity shown by the fractions retrieved after the first purification process.

Fraction	DPPH		Tyrosinase	
	Inhibition %	SD %	Inhibition %	SD %
1	97.7	0.53	37.4	3.69
2	59.6	5.16	12.7	8.75
3	56.8	7.05	11.0	1.74
4	3.43	1.76	20.8	15.7
5	91.2	4.40	28.6	7.30
6	50.8	3.75	0.03	0.06
7	96.4	0.74	78.6	0.74
8	95.6	0.40	76.9	5.42
9	98.8	0.55	55.2	1.32
**10**	**97.1**	**0.72**	**87.4**	**0.85**
11	97.5	0.66	65.2	1.16
12	22.1	5.35	6.35	1.07
13	97.5	0.33	35.8	4.45
14	96.8	0.64	77.2	0.19
15	90.0	9.19	75.6	2.32
16	89.0	9.91	28.1	1.71
17	16.9	4.39	0.00	0.00

Second Fractionation Activities

**Table 3:** Relative activity shown by the fractions retrieved after the second purification process.

Fraction	DPPH		Tyrosinase	
	Inhibition %	SD %	Inhibition %	SD %
10.1	28.6	13.2	35.8	4.45
10.2	18.0	5.07	57.1	0.90
**10.3**	**72.6**	**10.2**	**82.4**	**0.87**
10.4	35.3	22.7	52.5	3.35
10.5	32.0	15.3	51.9	3.14

Third Fractionation

**Table 4:** Relative activity shown by the fractions retrieved after the third purification process.

Fraction	DPPH		Tyrosinase	
	Inhibition %	SD %	Inhibition %	SD %
10.3.1	28.6	13.2	0.0	0.0
10.3.2	18.0	5.07	0.0	0.0
**10.3.3**	**72.6**	**10.19**	**8.44**	**1.59**
10.3.4	35.3	22.7	1.18	1.21
10.3.5	32.0	15.28	0.0	0.0

### 3.3. Molecular Characterization

Combining the three successive fractionation steps allowed us to obtain fraction 10.3.3 that was re-analyzed by analytical HPLC revealing a single peak with >99% UV-purity. Starting from 20 g of litchi dry roots, we obtained around 9 mg of fraction 10.3.3 (protocol yield of 0.045 g/Kg DM).

#### 3.3.1. IR

**Figure 5:**
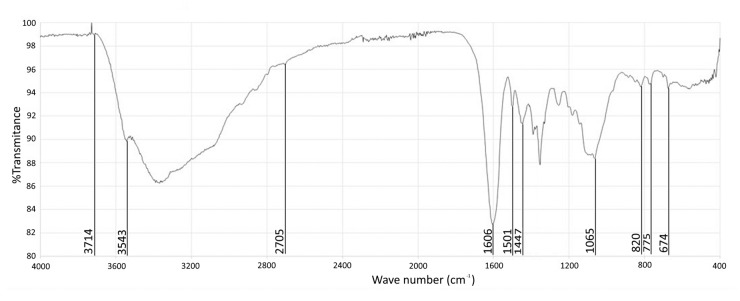
FTIR spectrum.

The most striking feature of the infrared spectrum (Figure 5) is the intense and relatively broad band around 1600 cm^−1^. Such an intense band is not expected from double bond stretching vibration but has been observed in molecules with 1,3-diketone moiety. The band is thought to result from the resonance and the formation of a strong hydrogen bond when one of the ketones enolizes. Carbonyl stretching bands are sometimes observed in 1,3-diketones infrared spectrum but the intensity of the bands is highly variable according to the molecular structure. 1,3-dihydroxy aromatic structures such as resorcinol and phloroglucinol also show an intense band around 1600 cm^−1^ and no intense band in the carbonyl region of the spectrum. This could suggest that the isolated molecule is a phenolic compound possessing −OH moieties in 1,3 positions. The occurrence of a small node at 3543 cm^−1^ suggests an intermolecular bonded −OH. The strong band at 1065 cm^−1^ can be explained by C-O stretching from ether bond.

#### 3.3.2. Mass Spectrometry

**Figure 6:**
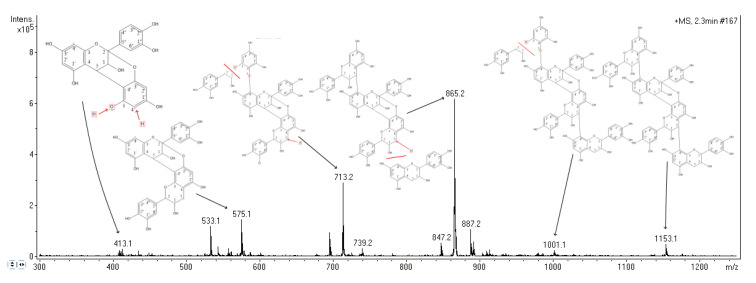
MS spectra of the purified molecule recorded in the positive mode.

Based on the mass spectrum (Figure 6), signal at 1153.1 m/z corresponded to [M + H]^+^, indicating a monoisotopic mass of 1152.1 Da for the purified molecule. When compared to the literature, the observed parent ion is in line with the observations of Lv Q. et al., 2015 and Xu X. 2010 [19,20] and suggests a proanthocyanidin tetramer with two B-type bonds and one A-type bond (Figure 8). The following information obtained from the mass spectrum was consistent with that conclusion. Signal at 1001.1 corresponded to the loss of an hydroxyvinylbenzenediol unit [M + H − 152]^+^, as previously described by Enomoto H. et al., 2020 [21]. The most abundant ion of the mass spectrum (865.2 m/z) was formed by quinone methide fission which caused the loss of the first epicatechin unit linked with a single B-type 5–4′ bond. The 865.2 m/z ion thus corresponded to an epicatechin trimer [M + H − 288]^+^. Sodium adduct of the latter is also reported at 887.2 m/z [M + Na − 288]^+^, as well as the formation of an ion resulting from a water molecule loss to the trimer at 847.2 m/z [M + H – 288 − H_2_O]^+^. The signal at 713.2 was produced following the loss of a hydroxyvinylbenzenediol unit from the epicathechin trimer [M + H – 288 − 152]^+^. Specific fragment ions at 575.1 and 865.2 allowed to elucidate the position of the A-type double 5–4’/2-O-3’ (Figure 8) as those fragments are produced when the A-type bond is placed between the second and the third epicatechin units [22,23].

#### 3.3.3. NMR

**Figure 7:**
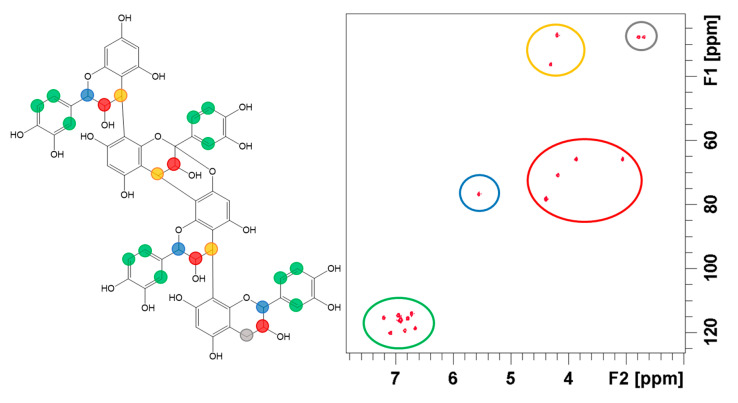
Two-dimensional heteronuclear single quantum correlation (HSQC) NMR spectrum of the isolated compound (right) and its hypothetical chemical structure (left). The structure proposal is based on both NMR and mass spectrometry data.

Based on its 2D ^1^H-^13^C HSQC spectrum (Figure 7), the isolated substance possesses at least eight unsaturated or aromatic C-H (green circle, Figure 7). The HSQC and HMBC spectra (Figures S1–S5) comparison revealed that the molecule also contains several unsaturated quaternary carbon atoms (154, 144, 131, 108, 106, 99 ppm). The quaternary C around 154 and 144 ppm are supposed to be linked to O in opposition to the 99 ppm and 130 ppm ones. The molecule does not seem to possess ketone, aldehyde or carboxylic acid moieties but includes an aliphatic part as suggested by several HSQC C-H signals (yellow, blue and red circles in Figure 7) and a CH_2_ signal (grey circle in Figure 7). From HSQC and HMBC data, this saturated structure is likely a heterocycle containing one oxygen atom. Several signals support the connection of the heterocycle to two aromatic structures: ^1^H at 5.55 ppm coupled to ^13^C at 115, 119 and 130 ppm; ^1^H at 2.8 and 2.7 ppm coupled to ^13^C at 154 and 100 ppm. A more detailed allocation of HMBC signals is provided in Supplementary Materials (Figure S6).

NMR data thus confirm that the molecule is a catechin derivative. This complies with the techniques used when isolating and purifying the compound [19,24] as well as when comparing to the available literature on the phytochemistry of Litchi [13].

If the catechin structure seems relevant, there are still too many signals corresponding to aromatic C-H, non-aromatic hydroxylated C-H and non-aromatic C-H. As suggested by mass spectrometry, several elements of NMR spectra support that the molecule is an oligomer, probably a tetramer from the repetitive patterns observed in HSQC and HMBC experiments:

- In HSQC, four signals are observed for non-aromatic hydroxylated C-H (red circle, Figure 7).
- In HMBC, four aromatic ^1^H (two around 6.9 ppm and two around 6.8 ppm) are also coupled to ^13^C with a common pattern (131; 144 ppm).
- In HSQC, the presence of signals at 4.2; 27 and 4.3; 36 ppm (Figure 7, yellow circle) can be explained if several catechin units are linked together since unpolymerized catechin should only display CH_2_ signals in this area of the spectrum.
- In HMBC, the B-type bond between units is confirmed by the coupling of ^1^H at 4.3 ppm with ^13^C at 154, 153, 151, 108, 106, 77 and 71 ppm. Signals at 4.3; 154,153 and 151 ppm correspond to H5 coupled to C1’, C3 and C3′. The coupling of H5 with C4 and C4′ are also expected and probably correspond to signals at 4.3; 108 and 106 ppm, respectively (HMBC spectrum in Figure S5).

**Figure 8:**
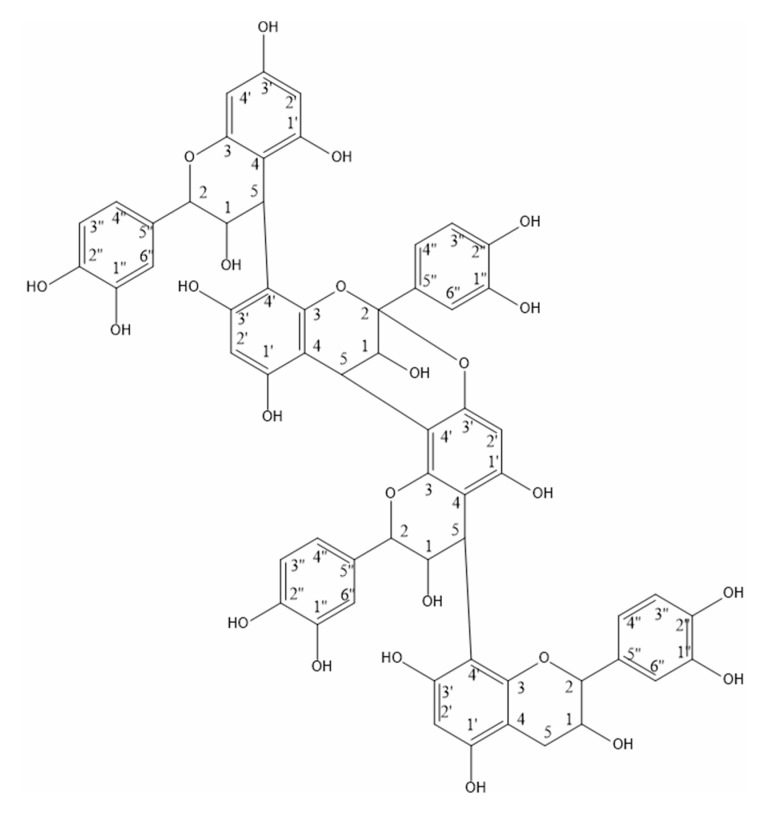
Purified compound from the roots of L. chinensis.

The presence of an A-type bond in the molecule is also confirmed by NMR data. Two distinct chemical shifts are attributed to carbon C2: 77–78 ppm and 99 ppm. The increased chemical shift (99 ppm) of carbon C2 (Figure 8) is consistent with its involvement in an A-type bond which implies an additional bond to an oxygen atom.

Using the NMR results coupled with IR results and the typical mass fragments, the isolated molecule is a proanthocyanidin composed of four catechins as seen in Figure 8.

#### 3.3.4. UV

As mentioned in the work of Hümmer & Schreier, 2008 [25], the presence of proanthocyanidin is characterized by a peak around 200–220 nm and a peak at 278 nm, this information is aligned with the UV spectra obtained from the isolated molecule (data not shown).

#### 3.3.5. Colorimetric Test

The Optical Density of the blanks and the sample were as follows (Table 5):

Based on the works of Q. Lv et al. (2015) M. J. Payne et al. (201) Y. Wang et al. (2016) [19,26,27], the compound reacts with DMAC, in a way that allows us to confirm the procyanidin nature of the compound as well as the presence of A linkage.

Finally, when comparing all the available information, the isolated compound is a A-type procyanidin tetramer with the following structure: EC -->B --> EC --> A -->EC --> B --> EC where EC stands for epicatechin, B stands for a single 5-4′ bond and A stands for a double 5-4′/2-O-3′ bond (Figure 8).

When comparing the determined structure to the available literature, a compound showing similar structure and mass is (2*R*,3*R*,4*S*,8*R*,14*R*,15*R*)-2,8-Bis(3,4-dihydroxyphenyl)-10-[(2*R*,3*R*,4*R*)-2-(3,4-dihydroxyphenyl)-3,4-dihydro-3,5,7-trihydroxy-2H-1-benzopyran-4-yl]-4-[(2*R*,3*S*)-2-(3,4-dihydroxyphenyl)-3,4-dihydro-3,5,7-trihydroxy-2H-1-benzopyran-8-yl]-3,4-dihydro-8,14-methano-2H,14H-1-benzopyrano[7,8-d][1,3]benzodioxocin-3,5,11,13,15-pentol or cinnamtannin D_2_ (CAS registry number 97233-47-1). This molecule was previously described in peanut skin (*Arachis hypogaea* L., Fabaceae; [28].

## 4. Conclusions

This work is part of a bigger study aiming to identify compounds of interest in the wide flora available in the Comoros archipelago. In this specific case, an anti-tyrosinase activity is interesting when willing to treat issues such as melasma [29]. This kind of skin condition implies the local application of compound is destined to regulate melanogenesis. Treatments used nowadays can be very dangerous as they often contain hydroquinone and steroids. Those compounds have shown adverse effects such as dermatitis, black and blue skin pigmentation, blindness, skin thinning, bruises and stretch marks. This means that there is room for effective treatment without any adverse effect especially when taken for a long period of time, as it is often requested for those types of diseases [30]. In addition, the antioxidant potency of the samples were tested, as it is also beneficial when working with topical treatments. Through a bio-guided purification followed by molecular characterization of extracts from the roots of *Litchi chinensis* Sonn., the structure of cinnamtannin D2 responsible for the biological activity was obtained. It is the first time that this class of compounds was highlighted in *L. chinensis’* roots. Lychee roots appear to be a promising source of raw material that could be used for the development of effective skin treatment. The fact that *L. chinensis* roots are used in this study may raise questions in terms of the plant’s conservation. However, technologies exist to enhance the value of root plant compounds [31]. In the end, our study has allowed the purification and the characterization of a proanthocyanidin A-type in *L. chinensis* roots for the first time. This study also revealed a consensus among the chemical structure, biological activities and the traditional uses of *Litchi* roots in Mayotte to treat skin pigmentation issues and soften the skin.

## Figures and Tables

**Table 1:** Main compounds found in L. chinensis with their location and identified biological activities [13].

Compound Class/Subclass	Compound	Plant Part	Biological Activity
Polyphenols		Leaves Seeds Pulp Pericarp	Cytotoxic Anti-viral Antioxidant Antimicrobial Lipid peroxidation inhibitory activities α-glucosidase inhibitory activities
Tannin	Coumarin	Seeds	Antioxidant
	Litchtocotrienol A-G Macrolitchtocotrienol A Cyclolitchtocotrienol A	Leaves	Cytotoxic
Lignan	Schizandriside	Leaves	Antioxidant
Lignan	Isolariciresinol	Pericarp	Antioxidant
Sesquiterpenes	Litchioside A and B Pumilaside A Funingensin A Pterodontriol-D-6-O-β-D-glucopyranoside	Seeds	Cytotoxic
Triterpenes		Aerial parts Seeds Pericarp	Antiviral
Sterols		Aerial parts Seeds Pericarp	Antiviral
Others	Litchiol A and B Secoisolariciresinol-9′-O-β-D-xyloside 4,7,7′,8′,9,9′-hexahydroxy-3,3′-dimethoxy-8,4′-oxyneolignan Ehletianol C Sesquipinsapol B, Sesquimarocanol B Ethyl shikimate, Methylshikimate Benzyl alcohol 5-(hydroxymethyl)furfural Hydrobenzoin	Leaves Pericarp Fruits	Antioxidant Cytotoxic

**Table 5:** Optical density at 640 nm of the fraction 10.3.3 in contact with 4-dimethylamincinnamaldehyde (DMAC).

Observation	MeOH	DMAC 1mg/mL	HCl + EtOH	Sample	Sample + DMAC
Optical density	0.00 ± 0.000	0.13 ± 0.00	0.00 ± 0.00	0.24 ± 0.00	0.46 ± 0.08

## Supplementary Materials

The following are available online at https://www.mdpi.com/2218-273X/10/9/1347/s1, Figure S1: 1D 1H NMR spectrum of the isolated compound, Figure S2: 2D HMBC NMR spectrum of the isolated compound, Figure S3: 2D HMBC NMR spectrum of the isolated compound, Figure S4: 2D HMBC NMR spectrum of the isolated compound, Figure S5: 2D HMBC NMR spectrum of the isolated compound, Figure S6: Allocation of 2D HMBC NMR signals of the isolated compound.
