# Supplementary material for: Identification of a Proanthocyanidin from Litchi Chinensis Sonn. Root with Anti-Tyrosinase and Antioxidant Activity
Source: Biomolecules. 2020 Sep 21;10(9):1347. doi: 10.3390/biom10091347 (PMC7565872; doi:10.3390/biom10091347)
Supplement: Supplementary file 1 [file biomolecules-10-01347-s001.zip › Supplementary material PDF.pdf]

Supplementary material

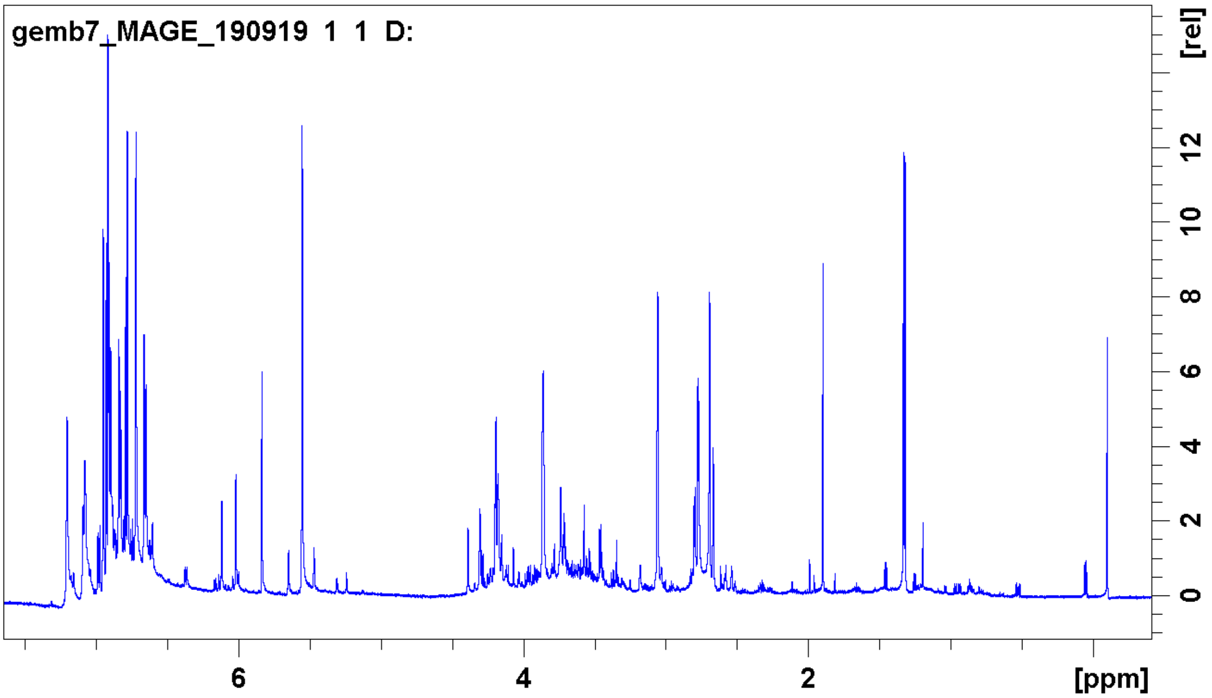

Supplementary figure 1 : 1D ^{1}H NMR spectrum of the isolated compound.

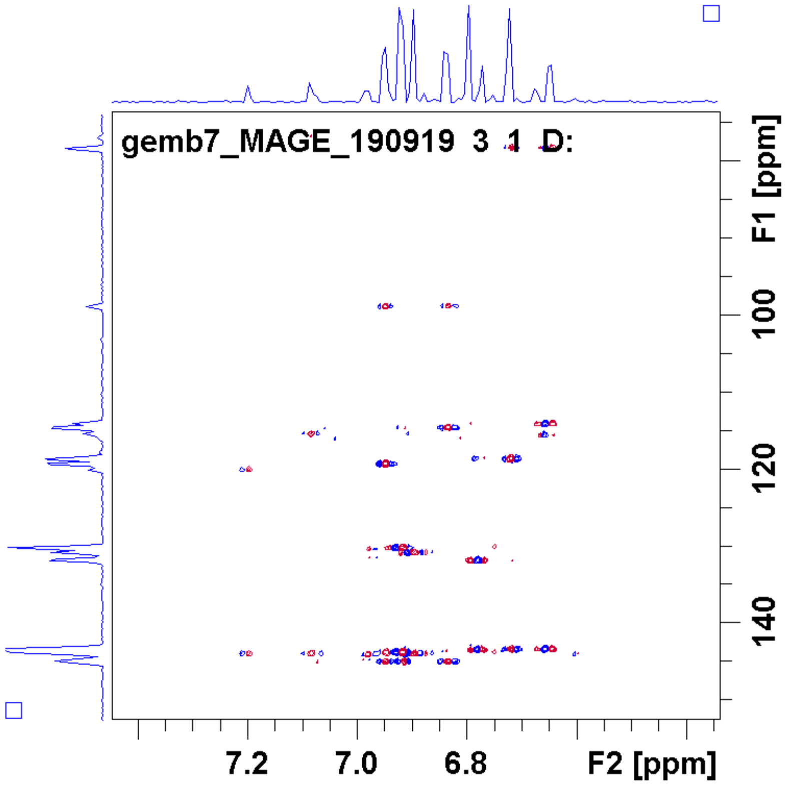

Supplementary figure 2: 2D HMBC NMR spectrum of the isolated compound.

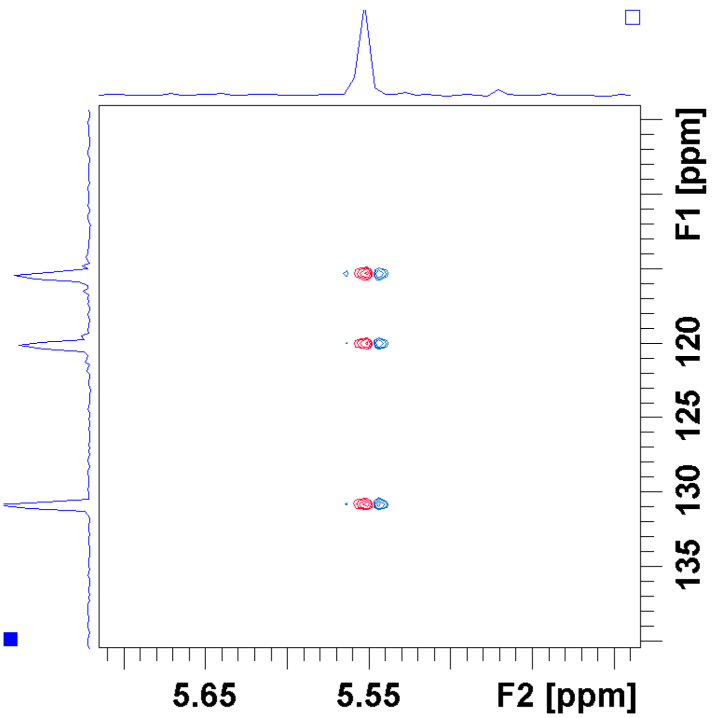

Supplementary figure 3 : 2D HMBC NMR spectrum of the isolated compound.

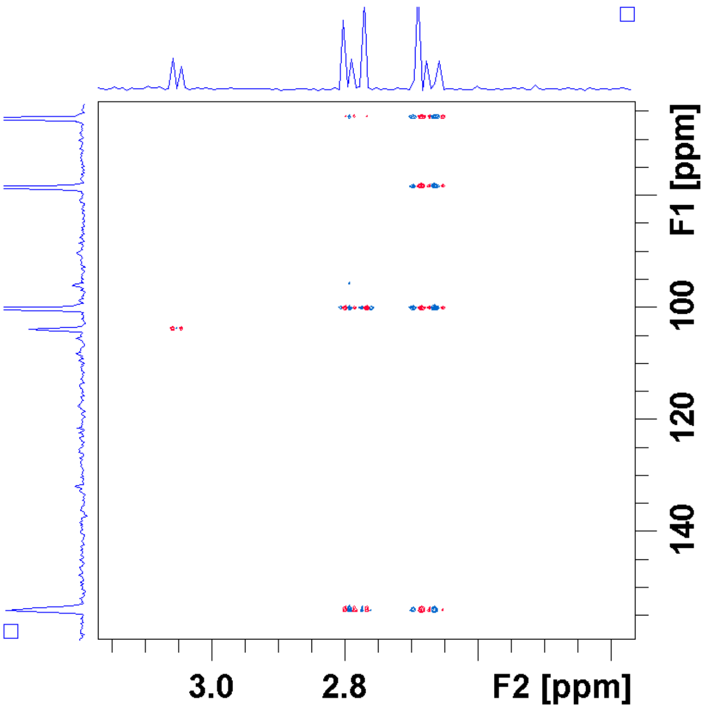

Supplementary figure 4 : 2D HMBC NMR spectrum of the isolated compound.

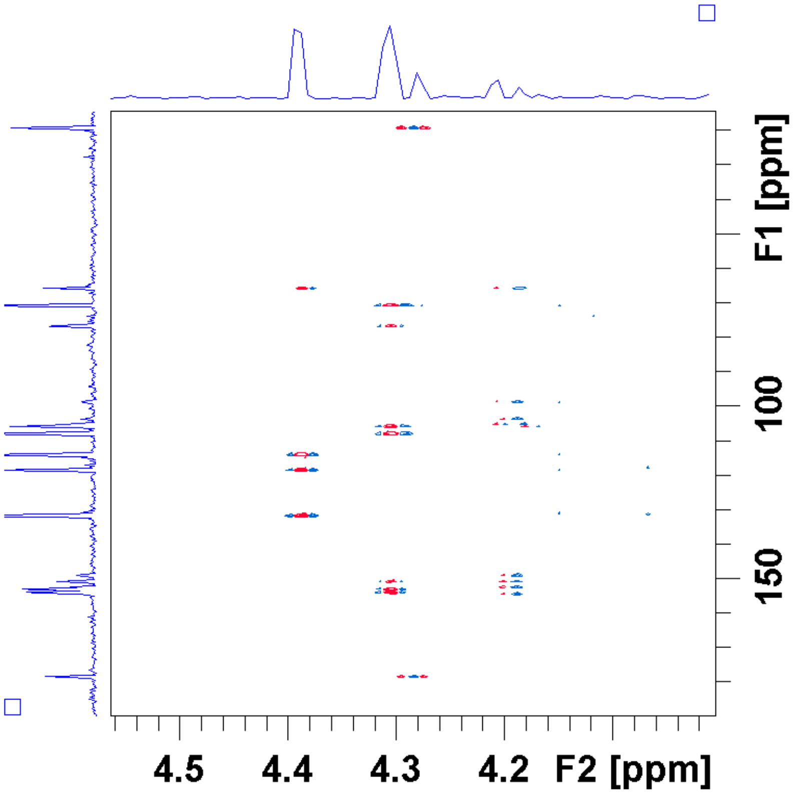

Supplementary figure 5 : 2D HMBC NMR spectrum of the isolated compound.

Line	Colour	^{1}H chemical shift (ppm)	$^{2}J_{CH}$ and $^{3}J_{CH}$ coupled ^{13}C chemical shift (ppm)
1		7.2	144,120,77
2		7.1	144, 116, 77
3		7.0	144, 119, 99
4		6.9	144, 131
5		6.9	144, 131
6		6.8	144,131
7		6.8	144, 131
8		6.8	144, 115, 99
9		6.7	144, 119, 78
10		6.6	144, 115, 78
11		5.8	105*
12		5.6	131, 120, 115
13		4.4	131, 119, 113, 66
14		4.3	154, 153, 151, 108, 106, 77, 71
15		4.2	149, 99, 66
16		3.9	100, 78
17		3.1	104
18		2.8	154, 100, 66
19		2.7	154, 100, 78, 66

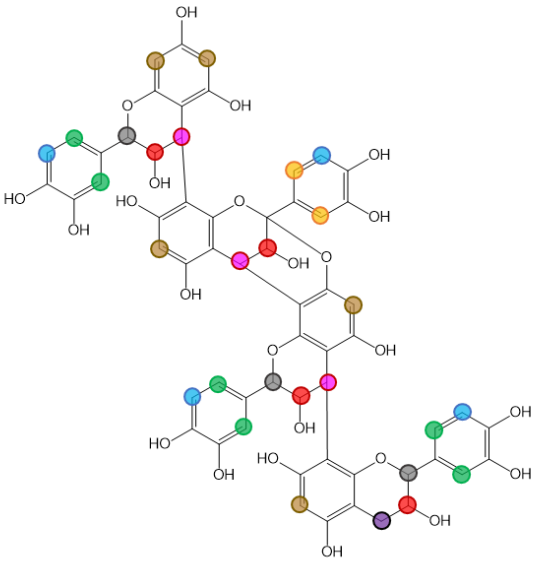

*Supplementary figure 6: allocation of 2D HMBC NMR signals of the isolated compound. Allocation colour indicates the position where the corresponding ^{1}H is attached to the structure. Chemical shifts were rounded leading to identical HMBC signals in some cases. * : this proton signal is perfectly visible in the 1D ^{1}H NMR spectrum and can be unambiguously attributed to an aromatic structure, however no corresponding C-H signal can be observed in 2D HSQC NMR spectrum. In HMBC, a very weak signal is observed.*
